# Supplementary material for: Comparative Transcriptomics of Multi-Stress Responses in Pachycladon cheesemanii and Arabidopsis thaliana
Source: Int J Mol Sci. 2023 Jul 11;24(14):11323. doi: 10.3390/ijms241411323 (PMC10379395; doi:10.3390/ijms241411323)
Supplement: Supplementary file 1 [file ijms-24-11323-s001.zip › Supplementary Figures.pdf]

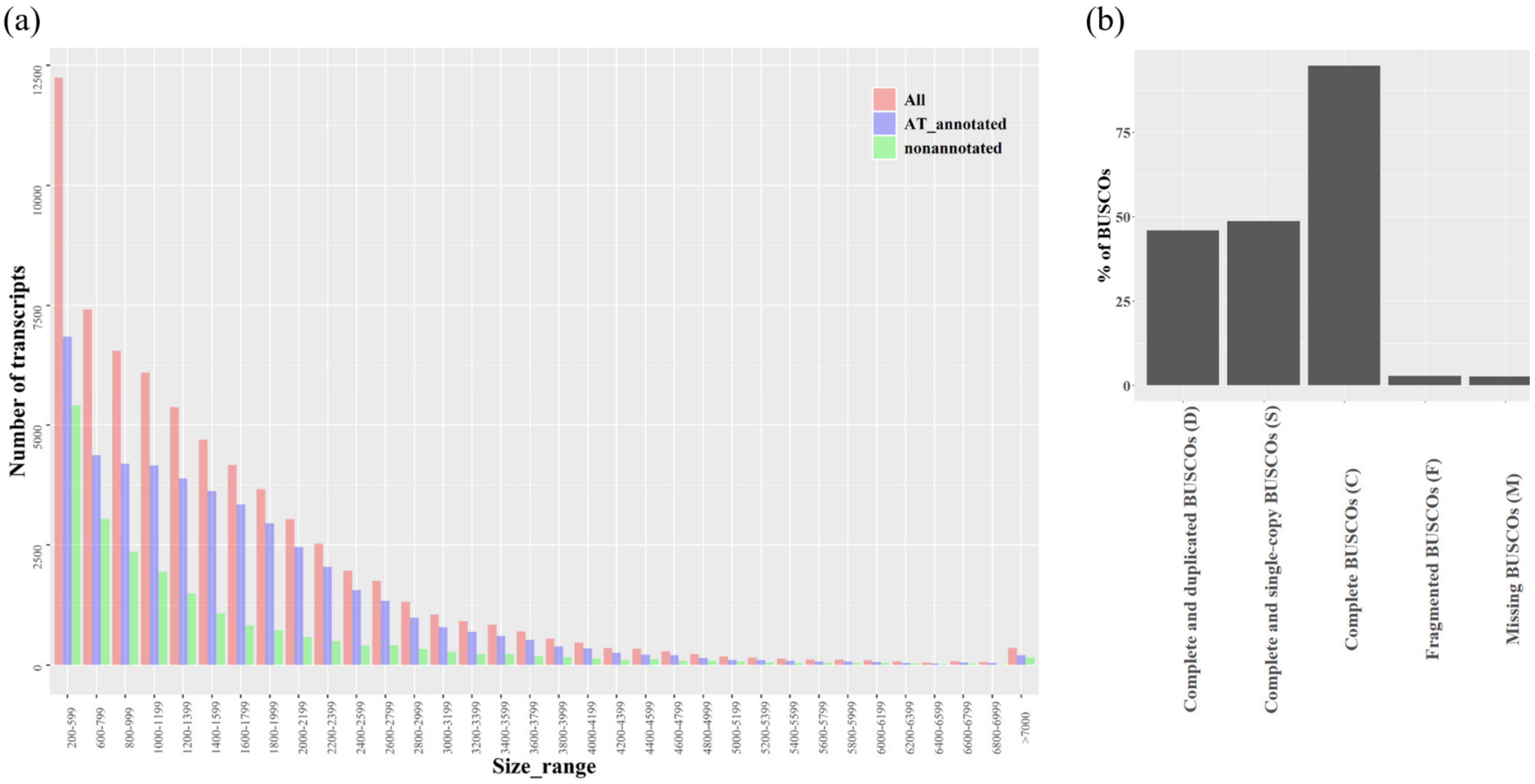

Figure S1 Length distribution of the assembled transcripts in *P. cheesemanii* and BUSCOs assessment of assembled transcriptome.

(a) length distribution of the assembled transcripts. Blue bar: the number of the transcripts could be annotated by *A. thaliana*; green bar: the number of the transcripts could not be annotated by *A. thaliana*. (b) BUSCOs assessment.

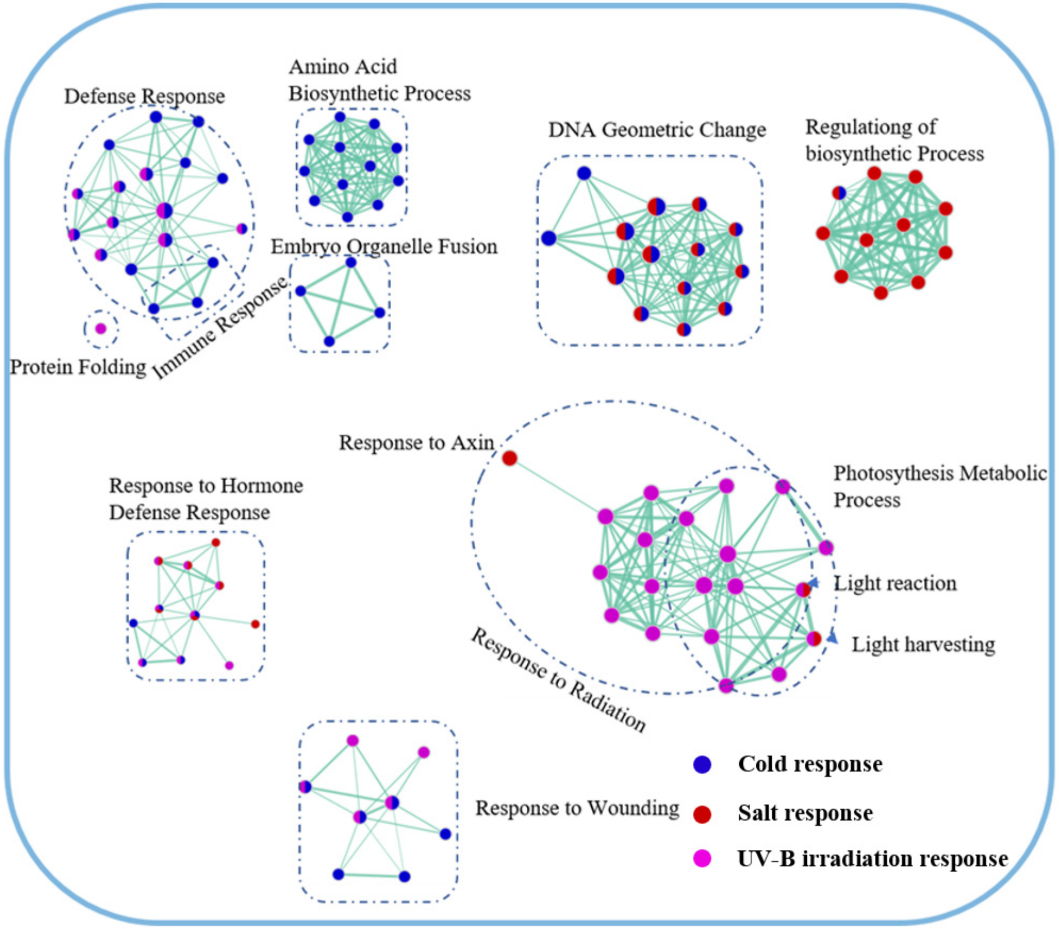

Figure S2 Network analysis of biological process of *A. thaliana* multiple stress-responsive transcriptomes in downregulation.

Purple circle: the overrepresented GO terms in UV-B radiation responses; red circle: the overrepresented GO terms in salt-induced responses; blue circle: the overrepresented GO terms in cold-induced responses. Box with dashed line: the cluster of the overrepresented GO terms involved in the same biological process.

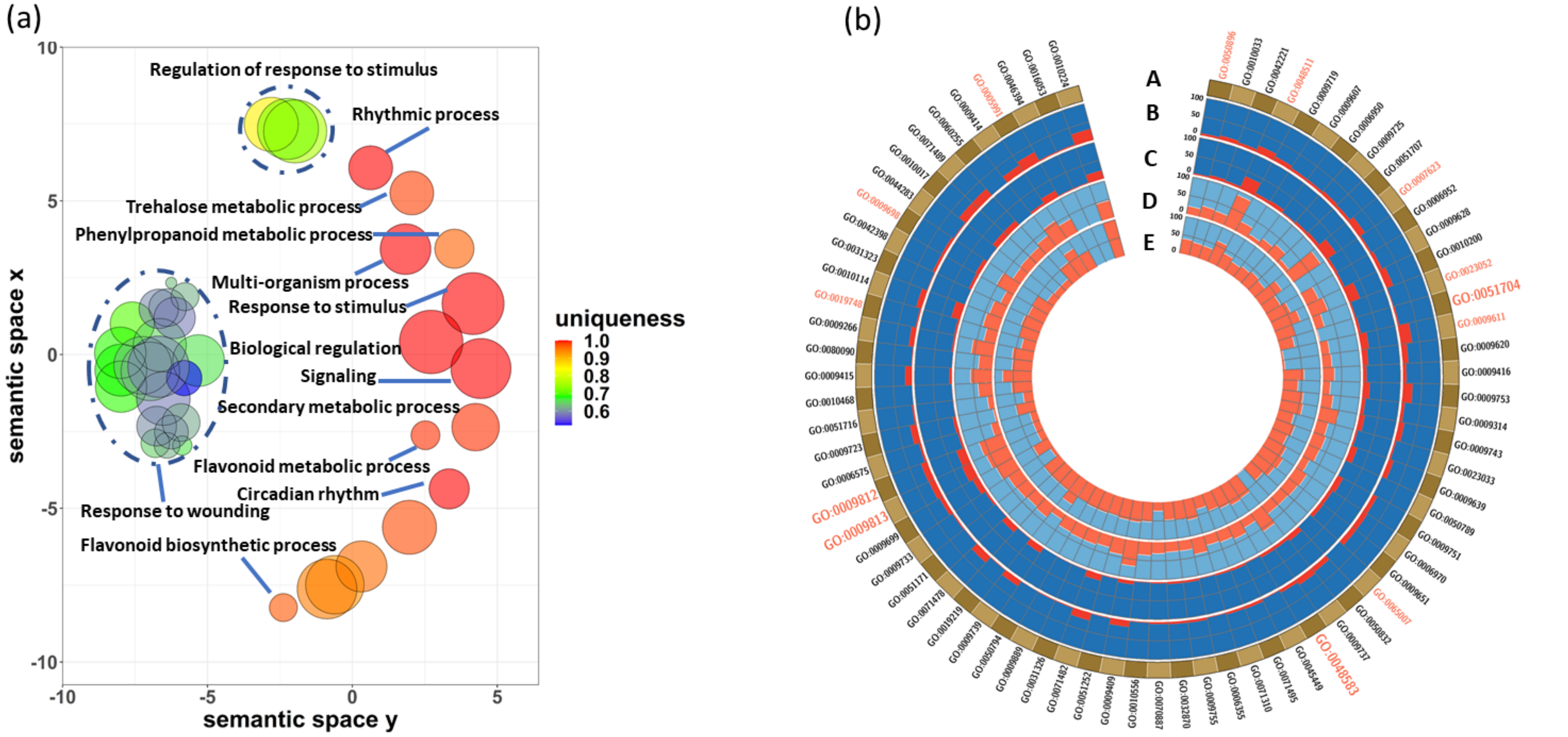

Figure S3 Summary of the common overrepresented terms of GO biological process of *A. thaliana* and *P. cheesemanii* in responding to cold stress.

(a) clustering of the common overrepresented terms of GO biological process of *A. thaliana* and *P. cheesemanii* in responding to cold stress. 74 shared overrepresented GO terms of *A. thaliana* and *P. cheesemanii* were clustered with 13 representatives of GO biological processes. Semantic space X and Y: no intrinsic meaning; uniqueness: measure whether the term is an outlier compared to the list. Namely, the negative of average similarity of a term to all other

terms. In REVIGO, multi-dimensional scaling was used to reduce the dimensionality of a matrix of the GO terms' pairwise semantic similarities. First, the terms were placed by using an eigenvalue decomposition of the terms' pairwise distance matrix. Then, a stress minimization step improved the agreement between the semantic similarities of the terms and their closeness in the two-dimensional space. Thus, the semantically similar GO terms should remain close together in the plot. Figure was generated from REVIGO web (http://revigo.irb.hr/) [30]. (b) comparisons of the induced genes and the rates of upregulated and downregulated genes in GO biological processes between *A. thaliana* and *P. cheesemanii* in responding to cold stress. Red labels: the representative GO biological processes in the left panel; enlarged red labels: the representative GO biological processes in the left panel with different rates of upregulated and downregulated genes. A, the common overrepresented GO biological processes of *A. thaliana* and *P. cheesemanii* in responding to cold stress; B, the percentage of induced genes out of the gene set of each GO term in *A. thaliana*; C, the percentage of induced genes out of the gene set of each GO term in *P. cheesemanii*; D, the percentage of upregulated and downregulated genes among the induced genes in *A. thaliana*; E, the percentage of upregulated and downregulated genes among the induced genes in *P. cheesemanii*.

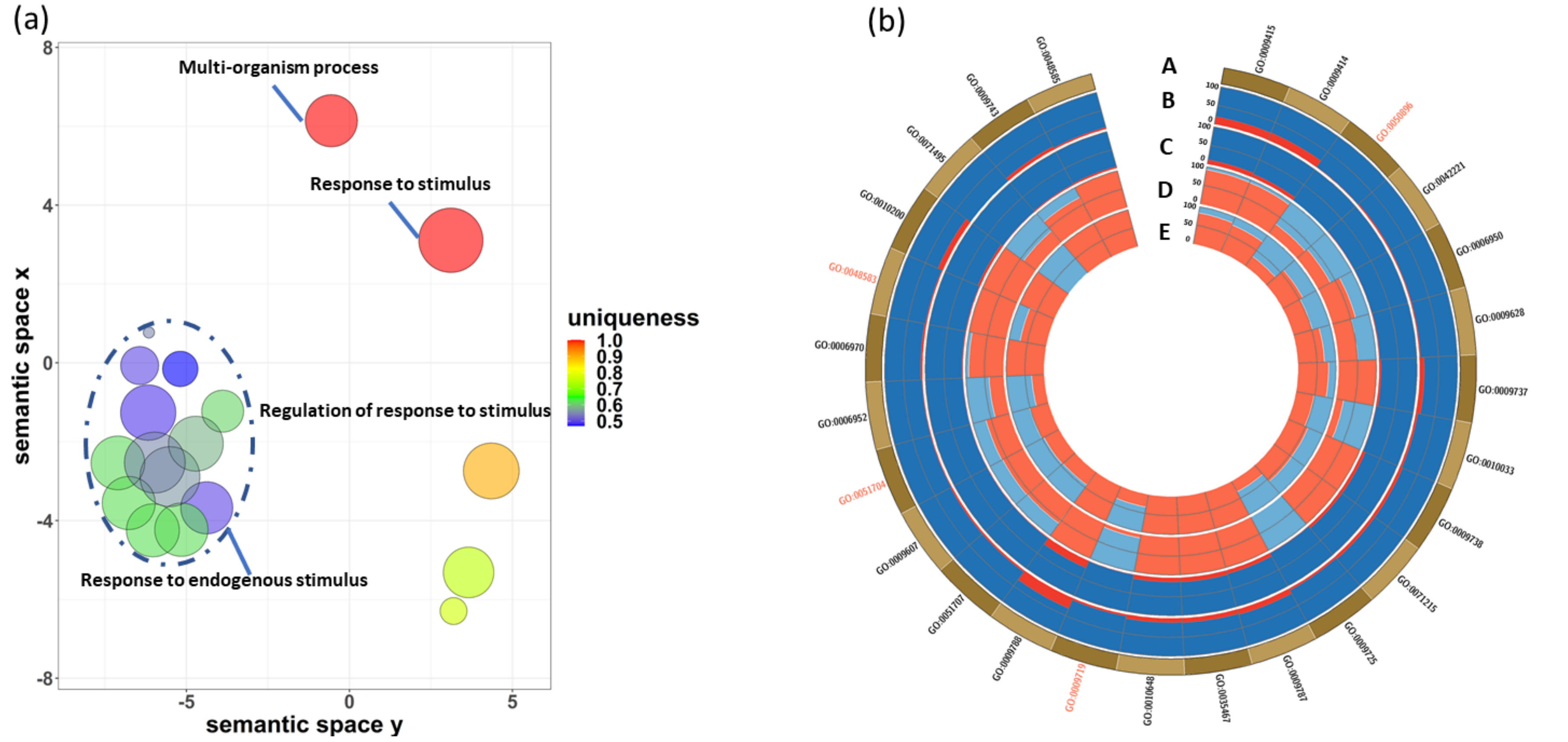

Figure S4 Summary of the common overrepresented terms of GO biological process of *A. thaliana* and *P. cheesemanii* in responding to salt stress.

(a) clustering of the common overrepresented terms of GO biological process of *A. thaliana* and *P. cheesemanii* in responding to salt stress. 26 shared overrepresented GO terms of *A. thaliana* and *P. cheesemanii* were clustered with 4 representatives of GO biological processes. Semantic space X and Y: no intrinsic meaning; uniqueness: measure whether the term is an outlier compared to the list. Namely, the negative of average similarity of a term to all other

terms. In REVIGO, multi-dimensional scaling was used to reduce the dimensionality of a matrix of the GO terms' pairwise semantic similarities. First, the terms were placed by using an eigenvalue decomposition of the terms' pairwise distance matrix. Then, a stress minimization step improved the agreement between the semantic similarities of the terms and their closeness in the two-dimensional space. Thus, the semantically similar GO terms should remain close together in the plot. Figure was generated from REVIGO web (http://revigo.irb.hr/) [30]. (b) comparisons of the induced genes and the rates of upregulated and downregulated genes in GO biological processes between *A. thaliana* and *P. cheesemanii* in responding to salt stress. Red labels: the representative GO biological processes in the left panel. A, the common overrepresented GO biological processes of *A. thaliana* and *P. cheesemanii* in responding to salt stress; B, the percentage of induced genes out of the gene set of each GO term in *A. thaliana*; C, the percentage of induced genes out of the gene set of each GO term in *P. cheesemanii*; D, the percentage of upregulated and downregulated genes among the induced genes in *A. thaliana*; E, the percentage of upregulated and downregulated genes among the induced genes in *P. cheesemanii*.

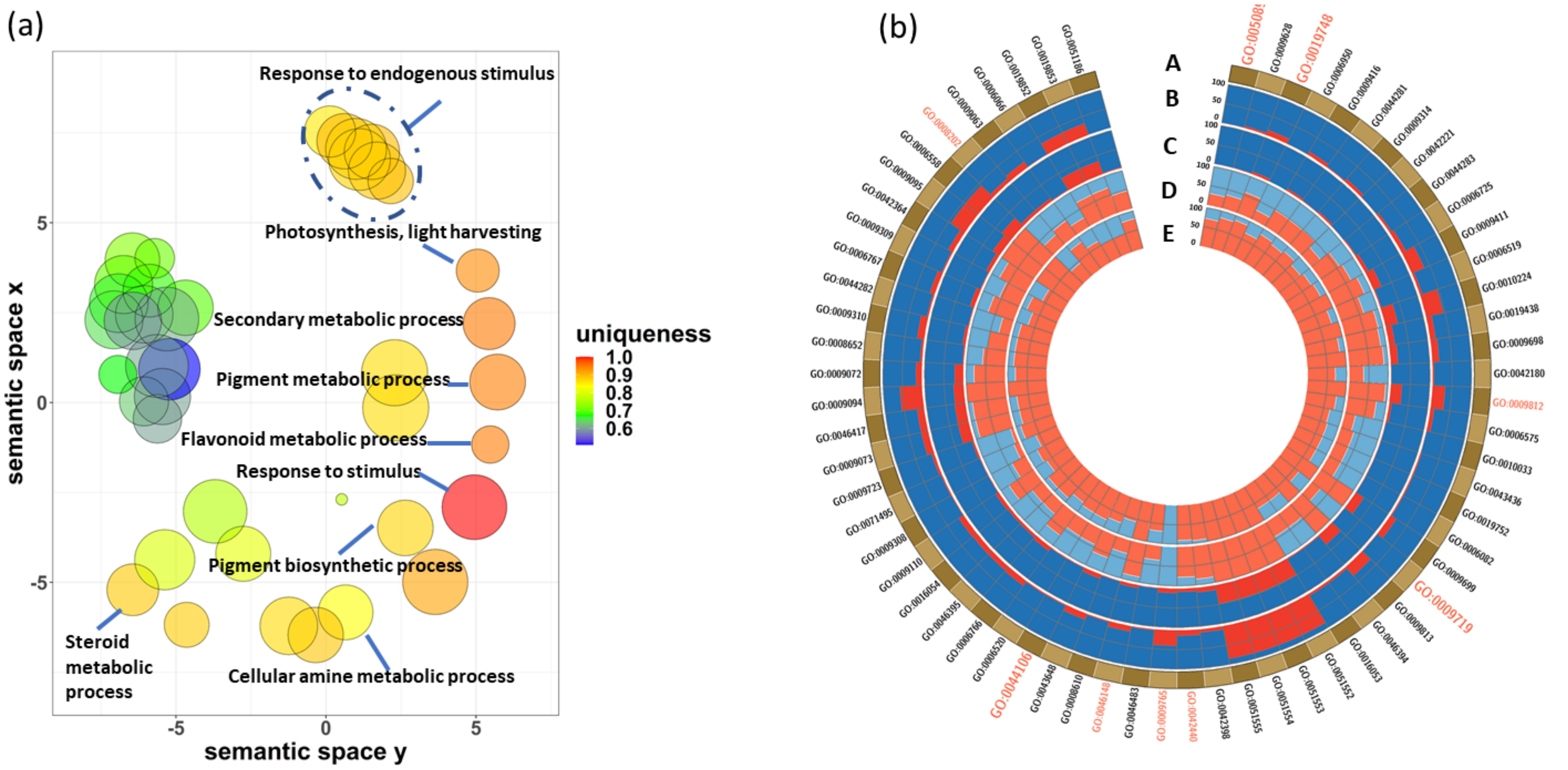

Figure S5 Summary of the common overrepresented terms of GO biological process of *A. thaliana* and *P. cheesemanii* in responding to UV-B radiation stress.

(a) clustering of the common overrepresented terms of GO biological process of *A. thaliana* and *P. cheesemanii* in responding to UV-B radiation stress. 65 shared overrepresented GO terms of *A. thaliana* and *P. cheesemanii* were clustered with 9 representatives of GO biological processes. Semantic space X and

Y: no intrinsic meaning; uniqueness: measure whether the term is an outlier compared to the list. Namely, the negative of average similarity of a term to all other terms. In REVIGO, multi-dimensional scaling was used to reduce the dimensionality of a matrix of the GO terms' pairwise semantic similarities. First, the terms were placed by using an eigenvalue decomposition of the terms' pairwise distance matrix. Then, a stress minimization step improved the agreement between the semantic similarities of the terms and their closeness in the two-dimensional space. Thus, the semantically similar GO terms should remain close together in the plot. Figure was generated from REVIGO web (http://revigo.irb.hr/) [30]. (b) comparisons of the induced genes and the rates of upregulated and downregulated genes in GO biological processes between *A. thaliana* and *P. cheesemanii* in responding to UV-B radiation stress. Red labels: the representative GO biological processes in the left panel; enlarged red labels: the representative GO biological processes in the left panel with different rates of upregulated and downregulated genes. A, the common overrepresented GO biological processes of *A. thaliana* and *P. cheesemanii* in responding to UV-B radiation stress; B, the percentage of induced genes out of the gene set of each GO term in *A. thaliana*; C, the percentage of induced genes out of the gene set of each GO term in *P. cheesemanii*; D, the percentage of upregulated and downregulated genes among the induced genes in *A. thaliana*; E, the percentage of upregulated and downregulated genes among the induced genes in *P. cheesemanii*.

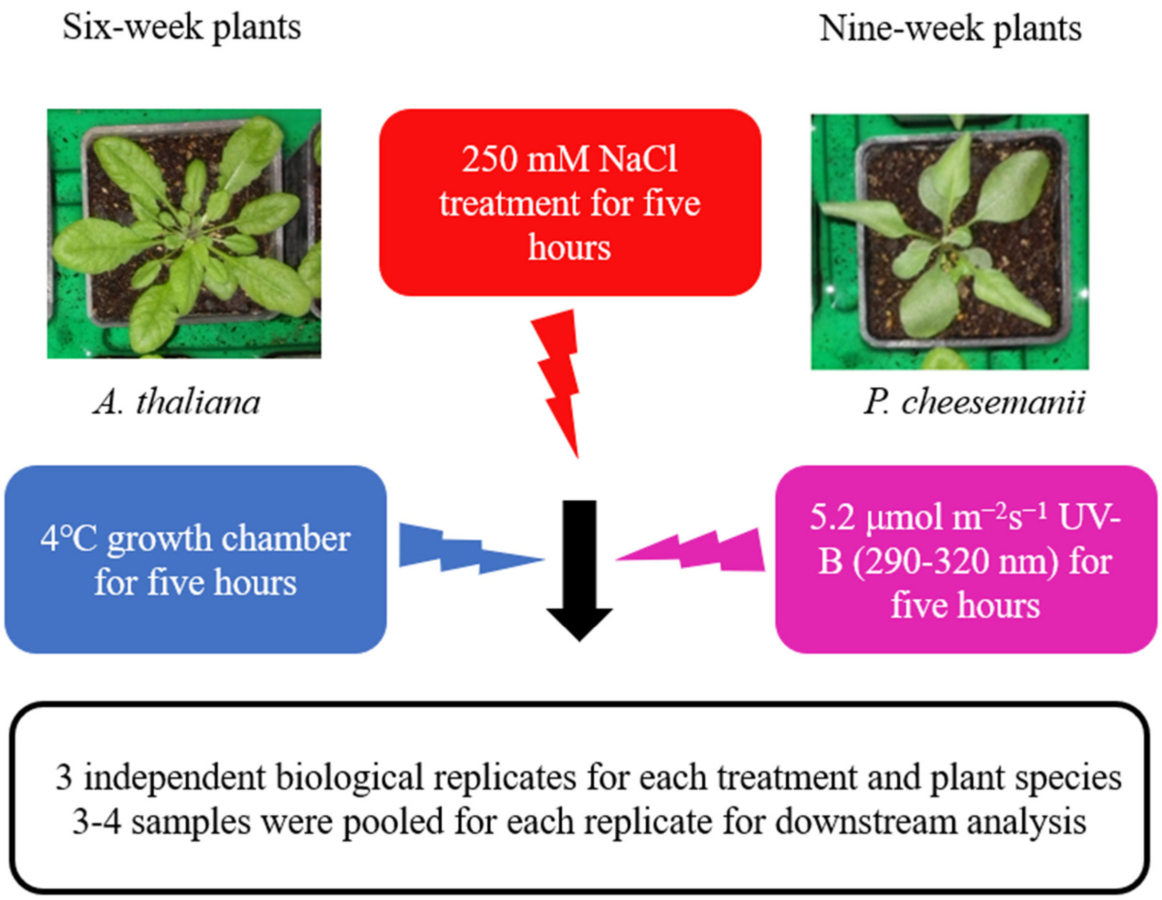

Figure S6 Five-hour stress treatment of six-week-old *A. thaliana* and nine-week-old *P. cheesemanii* plants for quantification of transcript abundance and multiple stress transcriptome profiling.

Six-week-old *A. thaliana* and nine-week-old *P. cheesemanii* plants after five-hour stress treatment were collected for quantification of transcript abundance and multiple stress transcriptome profiling. *A. thaliana* (six weeks old) and *P. cheesemanii* (9 weeks old) plants were grown in short-day condition and subsequently transferred to UV-B-supplemented white light, 4°C cold stress, 250 mM NaCl treatment for five hours or to white light only (control). Two mature leaves of each plant were collected after the treatments, and the leaves from three to four plants were pooled into an independent biological replicate. Three independent biological replicates for each treatment and plant accession were processed to the downstream quantification of transcript abundance and multiple stress transcriptome profiling.
