# Supplementary material for: Comparative Transcriptomics of Multi-Stress Responses in Pachycladon cheesemanii and Arabidopsis thaliana
Source: Int J Mol Sci. 2023 Jul 11;24(14):11323. doi: 10.3390/ijms241411323 (PMC10379395; doi:10.3390/ijms241411323)
Supplement: Supplementary file 1 [file ijms-24-11323-s001.zip › Supplementary File S3.pdf]

RNA extraction protocol

1. Fresh mature leaves were ground into the fine powder using mortar and pestle with liquid nitrogen. The ground samples were transferred into 1.7 mL tubes, and 1 mL of RNA Lysis Buffer was added to the ground tissue.

2. The tubes were vortexed for 20 seconds then kept on ice for 15 min. The centrifugation at 17000 g for 1 min was performed.

3. After centrifugation, 600 ul of the supernatant was transferred onto a Spin-Away^{TM} filter-column that was placed in a collection tube. Columns in collecting tubes were centrifuged at 17000 g for 30 s and were discarded afterward.

4. The same volume (500 ml) of ethanol was added into the flow-through in collecting tubes.

5. The mixture was transferred into a Zymo-Spin^{TM} IIICG column, which was placed in a new collection tube, and centrifuged at 17000 g for 30 s.

6. Zymo-SpinTM IIICG columns with bound RNA were washed with 100 μl of RNA wash buffer centrifuging at 17000 g for 30 s and the flow-through was discarded.

7. 50 μl of DNase mixture (2 μl of DNase (Roche, USA), 5 μl of 10X DNase buffer, 43 μl of water from the kit) was added on the matrix of each Zymo-SpinTM IIICG column, and the tubes with Zymo-SpinTM IIICG columns were placed on a heat block at 37°C for 15 min.

8. The columns were washed with 400 μl of RNA Prep buffer, 700 μl of RNA Wash buffer, and 400 μl of RNA Wash buffer centrifuging at 17000 g for 30 s.

9. Then, Zymo-SpinTM IIICG columns were placed in 1.5 mL tubes. 30 μl of DNase/RNase free water was added to each column with (provided in the kit) followed by centrifugation at 17000 g for 1 min.

10. The final RNA samples were stored at -80°C.

RNA quality check

Quality and concentration of RNA samples were checked for 2 µl sample aliquots with NanoDrop™ 1000 spectrophotometer (Thermo Fisher Scientific, USA). Only samples with 260 nm/280 nm absorbance ratio > 1.8 and 260 nm/230 nm absorbance ratio > 1.5 were used for the further analysis.

RNA samples for the sequencing experiments were checked by using Agilent 2100 Bioanalyzer with RNA 6000 Nano kit (Agilent Technologies, USA) at Massey Genome Service. The samples with RNA integrity number (RIN) >7 were used for the further analysis.

cDNA synthesis

Transcriptor First Strand cDNA Synthesis Kit (Roche, USA) has been used to synthesize cDNA. 1 µg RNA sample were transferred into a 200 µl PCR tube, followed by adding 1 µl of Anchored-oligo (dT18) primers and water (provided with the kit). The final volume was 13 µl. Mixtures were kept at 65°C for 10 min in Mastercycler® Pro machine (Eppendorf, Germany). Then, 0.5 µl of RNase inhibitor solution, 0.5 µl of reverse transcriptase enzyme solution, 2 µl of deoxynucleotide mix, and 4 µl of 5X reaction buffer in total 7 ul were added into each previous mixtures which made 20 ul final reaction volumes. The reactions were further incubated at 55°C for 30 min then terminated by 85°C incubation for 5 min. The synthesized cDNA was stored at -20°C.
