# Supplementary material for: Comparative Transcriptomics of Multi-Stress Responses in Pachycladon cheesemanii and Arabidopsis thaliana
Source: Int J Mol Sci. 2023 Jul 11;24(14):11323. doi: 10.3390/ijms241411323 (PMC10379395; doi:10.3390/ijms241411323)
Supplement: Supplementary file 1 [file ijms-24-11323-s001.zip › Supplementary Tables.pdf]

Table S1. Summary of read data for *P. cheesemanii* stress transcriptome.

Library	Raw reads	Clean reads	Raw data (GB)	Clean data (GB)
**Control**	115,407,011	115,383,228	32.24	32.24
**UV-B**	99,224,069	99,204,290	27.72	27.72
**Cold**	111,242,384	111,202,228	31.08	31.07
**Salt**	111,282,532	111,236,246	31.09	31.08
**Total**	437,155,996	437,025,992	122.13	122.11

Table S2. Assessment of transcriptome assemblies generated by multiple assemblers.

	k-mer size	Bowtie (%)	Complete BUSCOs (%)	Fragmented BUSCOs (%)	Missing BUSCOs (%)
	k55	88.21	92.7	4.5	2.8
	k65	87.84	96.1	1.9	2
**Velvet/Oases**	k75	87.56	97.1	1.3	1.6
	k85	86.64	96.9	1.4	1.7
	k95	85.9	96.4	2.1	1.5
	k51	90.79	92.5	4.4	3.1
	k53	90.88	92.8	4.4	2.8
	k55	90.98	93.1	4	2.9
**Trans-ABySS**	k57	91.08	93.8	3.5	2.7
	k59	91.4	94.3	3.5	2.2
	k61	91.21	94	3.5	2.5
	k63	91.25	94.1	3.5	2.4
	k19	88.81	59.9	24.4	15.7
	k21	88.72	84.5	9.4	6.1
	k23	88.62	89.4	7.2	3.4
**Trinity**	k25	88.26	86.3	8	5.7
	k27	88.68	87	7.4	5.6
	k29	89.41	88.1	8.1	3.8
	k31	89.86	88.6	7.1	4.3

Table S3. Summary statistics for transcriptome assembly.

Assembly	
Trans-ABySS transcripts (combined 7 assemblies)	318,111
CAP3 transcripts	223,341
EvidentialGene transcripts	67,905
EvidentialGene genes	45,911
Minimum transcript length (bp)	200
Longest transcript length (bp)	18,443
Final size of assembly (bp)	107,064,440
